# Supplementary material for: Immature excitatory neurons develop during adolescence in the human amygdala
Source: Nat Commun. 2019 Jun 21;10:2748. doi: 10.1038/s41467-019-10765-1 (PMC6588589; doi:10.1038/s41467-019-10765-1)
Supplement: Supplementary file 1 — Supplementary Information [file 41467_2019_10765_MOESM1_ESM.pdf]

**Supplementary Information for Sorrells et al.**

**Immature excitatory neurons develop during adolescence in the human amygdala**

**Supplementary Figures 1–8**

**Supplementary Table 1: Case List**

**Supplementary Table 2: Antibodies**

**Supplementary Table 3: Genes differentially expressed in immature PL neurons in ASD**

**Supplementary Data 1: Cell metadata for the amygdala snRNA-seq dataset**

**Supplementary Data 2: Mixed linear models**

**Supplementary Data 3: List of markers of immature PL neurons identified by unbiased analysis of snRNA-seq data**

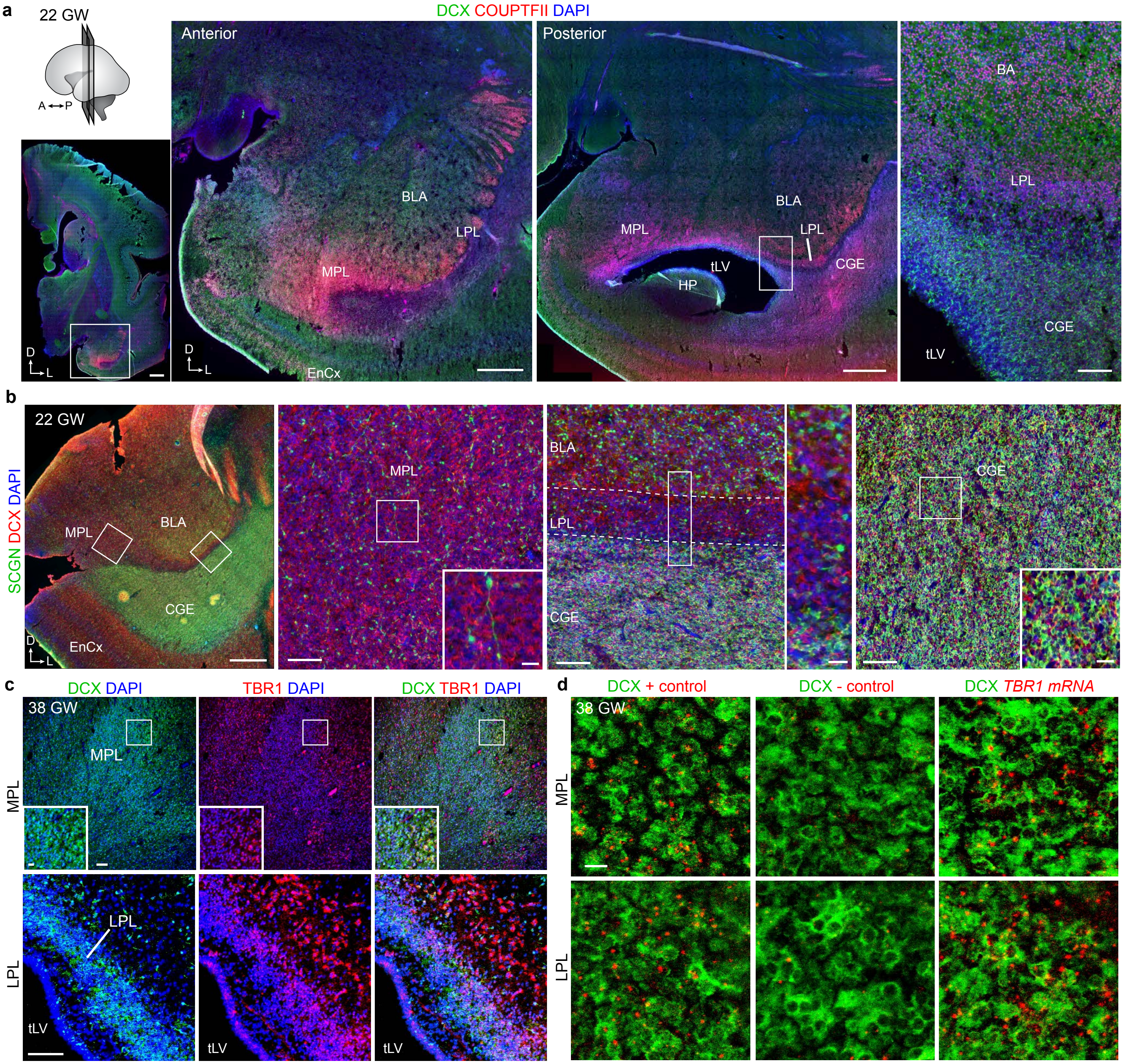


**Supplementary Figure 1: DCX⁺COUP-TFII⁺ cells expressing *TBR1* in the PL at 22 GW. a,** Coronal sections at 22 GW of the anterior (left) and posterior (right) human amygdala separated by 1 mm. DCX⁺COUP-TFII⁺ cells are present in the medial and lateral PL regions (MPL and LPL respectively) and in the CGE. **b,** (Left) SCGN⁺DCX⁺ cells in the temporal lobe at 22 GW. The MPL (Middle left) and LPL (Middle right) contain few of these cells compared to the BLA or CGE (Right). **c,** DCX⁺TBR1⁺ cells in the MPL and LPL at 38 GW. **d,** Single-molecule detection of *TBR1* mRNA (or the positive control PPIB) in the DCX⁺ cells within the MPL and LPL at 38 GW. Scale bars: 2 mm (**a** lower left), 1 mm (**a** middle left, **a** middle right, **b** left), 100 µm (**b** middle left, **b** middle right, **b** right, **c**), 20 µm (**b** insets, **c** insets), 10 µm (**d**).

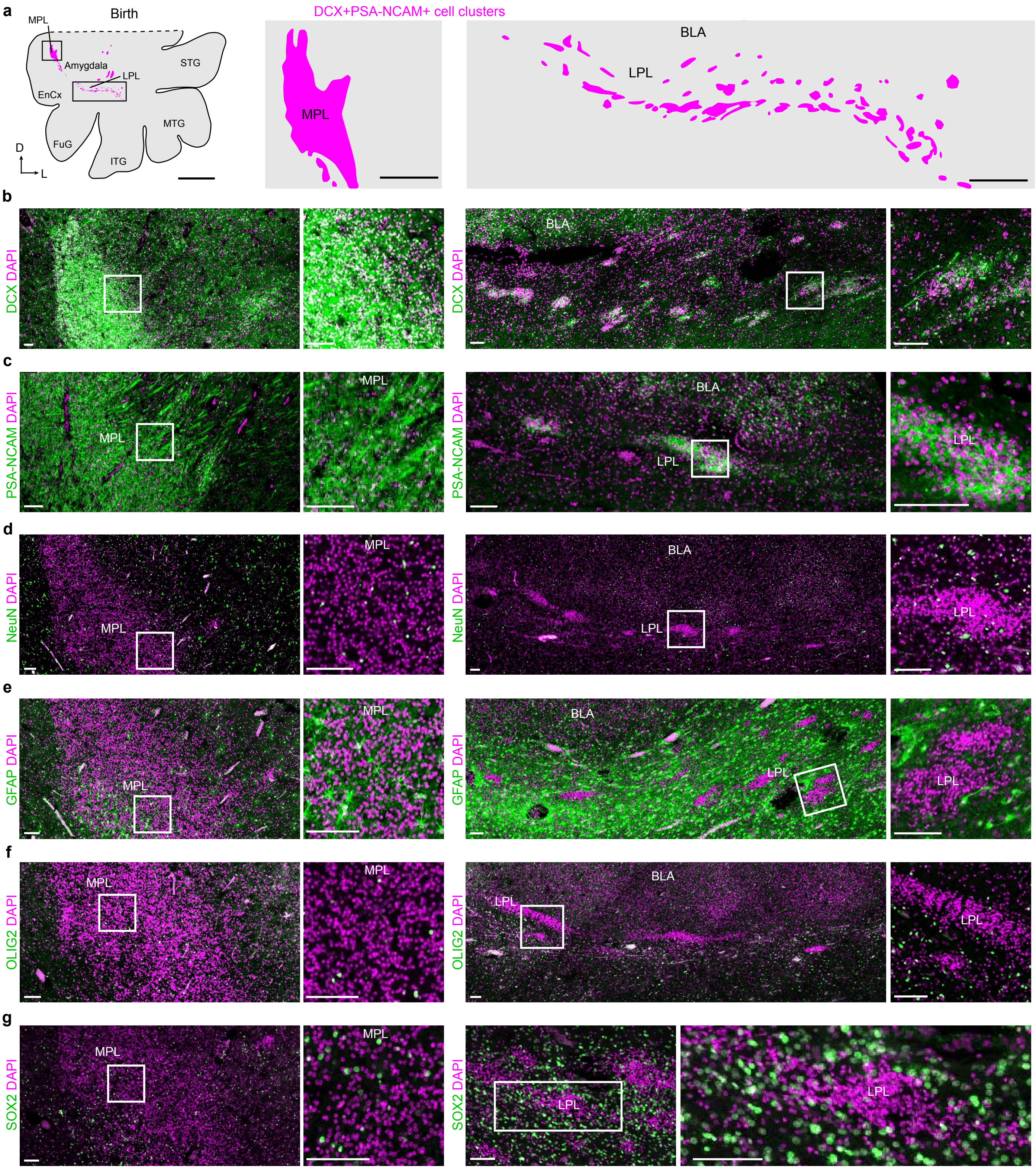


**Supplementary Figure 2: DCX⁺PSA-NCAM⁺ young neurons in the PL at birth. a,** Diagram of human temporal lobe at birth indicating clusters of DCX⁺PSA-NCAM⁺ cells in the MPL and LPL (higher magnification of **Fig. 3e** section i). (Right) MPL and LPL clusters correspond to regions stained in (**b–g**). **b,** DCX⁺ cells in the MPL and LPL clusters at birth. **c,** PSA-NCAM⁺ cells and fibers in the MPL and LPL at birth. **d,** NeuN⁺ cells in the MPL and LPL at birth. **e,** GFAP⁺ cells within the MPL and surrounding the LPL at birth. **f,** OLIG2⁺ cells in the MPL and LPL, and in the white matter surrounding the LPL at birth. **g,** SOX2⁺ cells in the MPL and LPL and surrounding the LPL at birth. Scale bars: 5 mm (**a** left), 500 μm (**a** middle, **a** right), 100 μm (**c**).

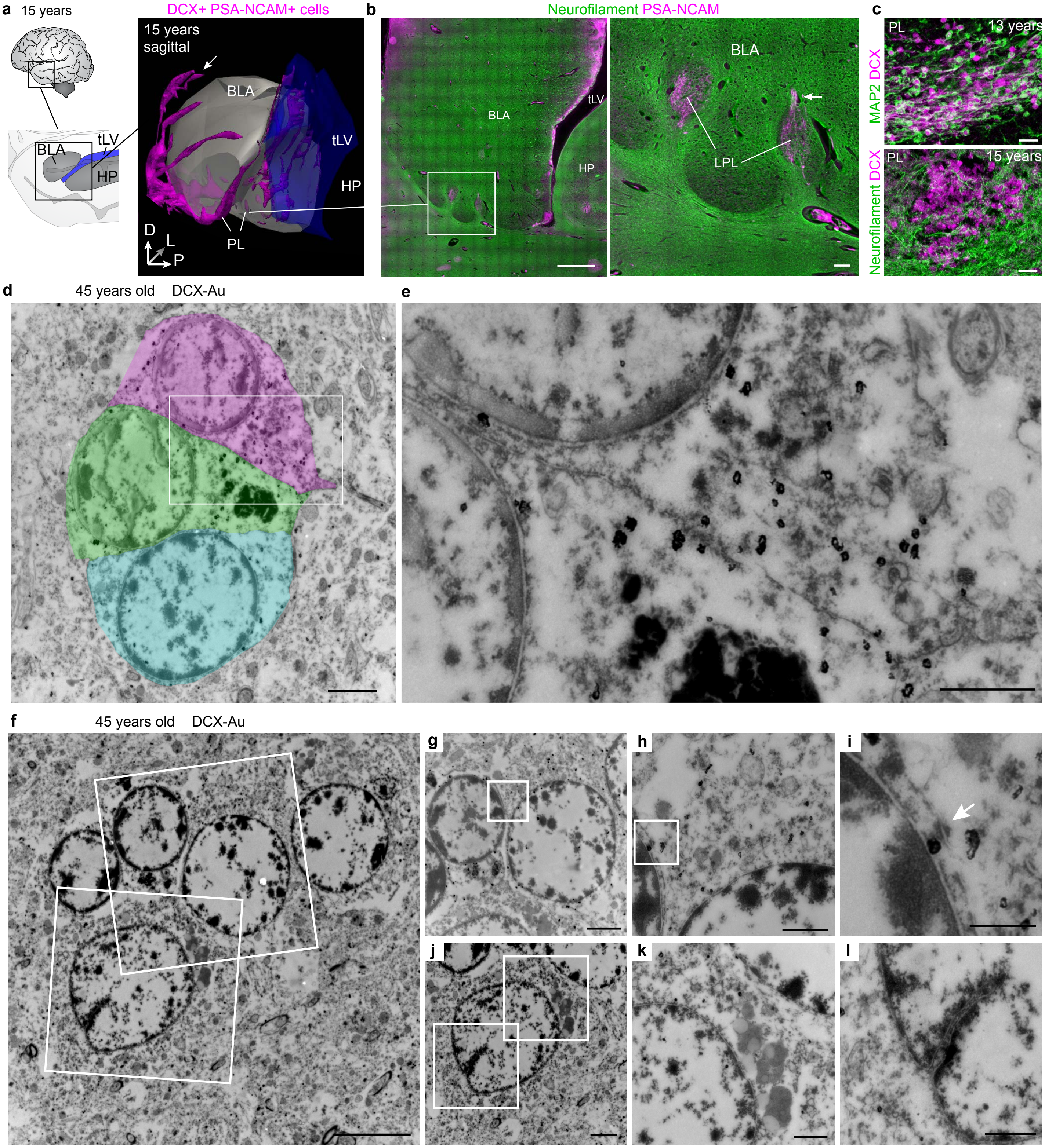


**Supplementary Figure 3: Connectivity of DCX⁺ cells in the PL.** **a,** (Left) Diagram of human brain at 15 years showing sagittal perspective of the temporal lobe. (Right) Sagittal view of a 3-D reconstruction of the right temporal lobe showing the location of DCX⁺PSA-NCAM⁺ cell clusters in the PL surrounding and protruding into the BLA at 15 years of age. The location of the intercalated cell nuclei is indicated (arrow). **b,** Sagittal section through the BLA at the level of the two LPL clusters indicated in (a). Staining for neurofilament and PSA-NCAM revealed fibers extruding from the angular bundle and wrapping around the LPL clusters. PSA-NCAM⁺ and neurofilament⁺ fibers are present on the dorsal side of the LPL cluster on the right (arrow). **c,** (Top) A subset of the DCX+ cells in the LPL at 13 years are MAP2⁺. (Bottom) neurofilament+ fibers surround and extend into the LPL at 15 years. **(d–l),** The ultrastructural analysis of DCX+ cells at 45 years of age detected small clusters of cells, similar to those observed by fluorescence microscopy, with features of immature neurons. At higher magnification most of these cells have small nuclei and their cytosol contains few

organelles, although they display a high ribosome density (**g–i**). We occasionally found adherens junctions between these cells, a hallmark of immature neurons that are in contact (arrow). More rarely, we found cells in these clusters with a phenotype more similar to mature neurons: a higher variety of organelles, accumulation of lipofuscin and even nuclear invaginations (**j–l**). Scale bars: 2 mm (**b** left), 200 μm (**b** right), 20 μm (**c**), 5 μm (**f**), 2 μm (**d**, **g**, **j**), 1 μm (**e**, **h**, **k**, **l**), 500 nm (**i**).

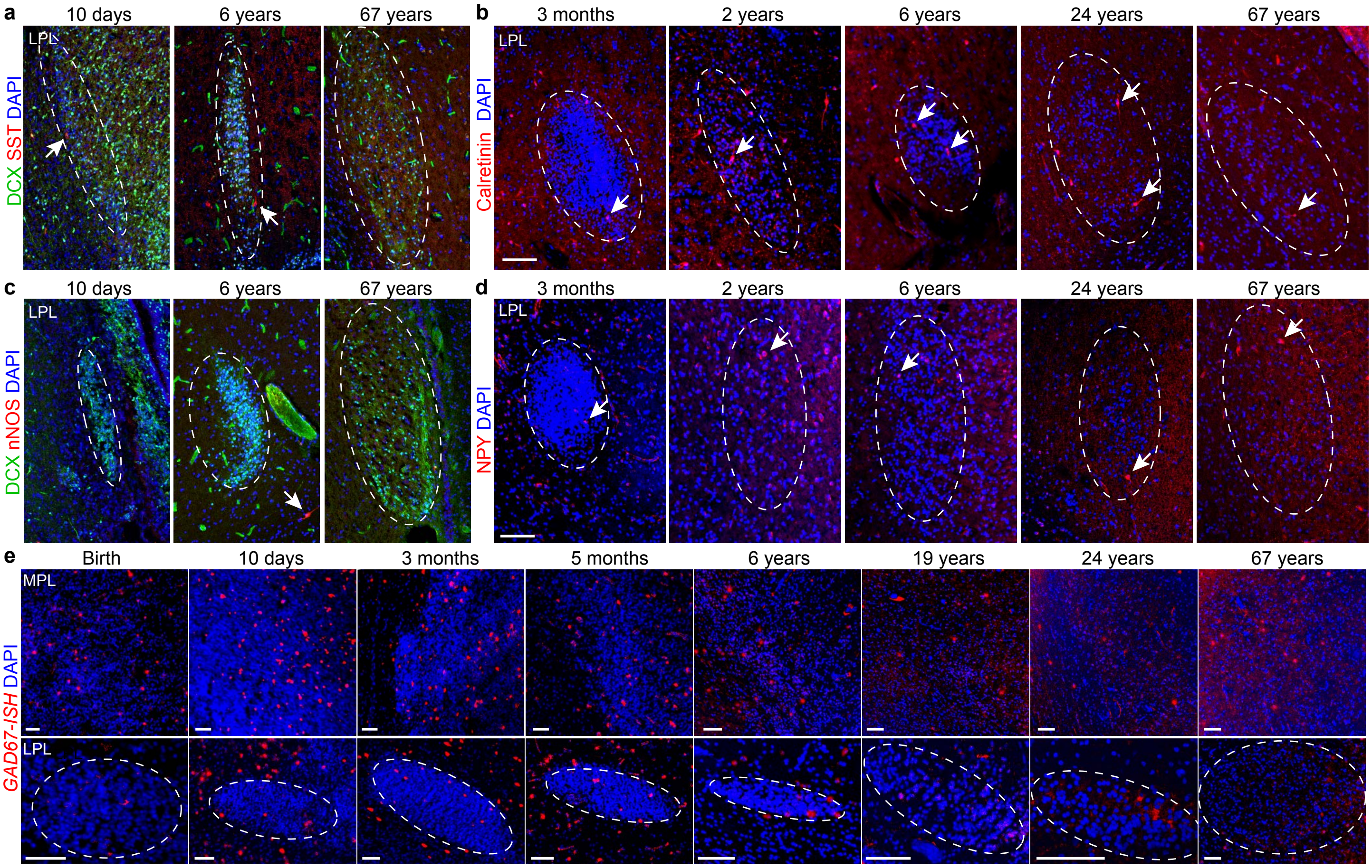


**Supplementary Figure 4: Interneurons in the PL throughout life. a,** SST⁺ cells (arrows) near the DCX⁺ cells in the LPL at 10 days and 6 years. **b,** Calretinin⁺ cells (arrows) in the LPL from 3 months to 67 years. **c,** Infrequent nNOS⁺ cells (arrow) near the DCX⁺ cells in the LPL at 10 days, 6 years, and 67 years. **d,** NPY⁺ cells (arrows) in the LPL from 3 months to 67 years. **e,** In situ hybridization for human *GAD67* in the MPL and LPL from birth to 67 years of age. Scale bars: 100 μm (**a–e**).

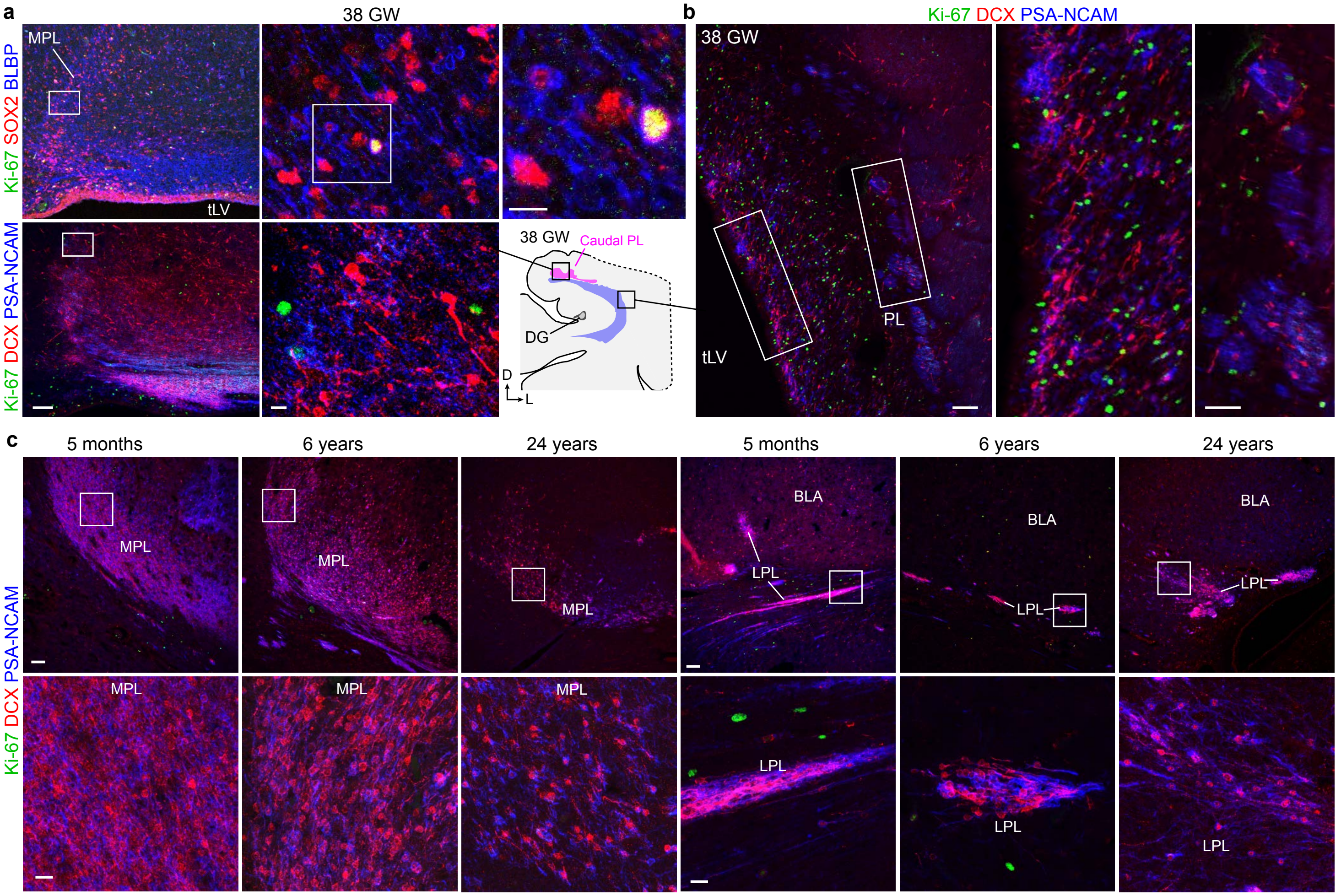


**Supplementary Figure 5: DCX⁺PSA-NCAM⁺ cells are Ki-67⁻ in the postnatal PL. a,** (Top) Ki-67⁺SOX2⁺BLBP⁺ cell in the MPL at 38 GW at the location in the caudal MPL indicated in the diagram in the lower right (the former site of high Ki-67+ cell density during gestation, see **Fig. 1a,b**. Coronal section level corresponds to (vi) from **Fig. 2a**.) (Bottom) Ki-67+ cells near DCX+PSA-NCAM+ cells in an adjacent section. **b,** Ki-67+ cells near DCX+PSA-NCAM+ cells in the lateral wall of the temporal lobe lateral ventricle (tLV) (Middle) and the caudal LPL (Right). **c,** Ki-67⁺ and DCX⁺PSA-NCAM⁺ cells in the MPL (Left) and LPL (Right) from 5 months to 24 years of age in the sections used to make the maps in (**Fig. 6a**). Scale bars: 100 μm (**a** left, **b** left, **c** top), 50 μm (**b** insets), 20 μm (**c** bottom), 10 μm (**a** middle, **a** top right).

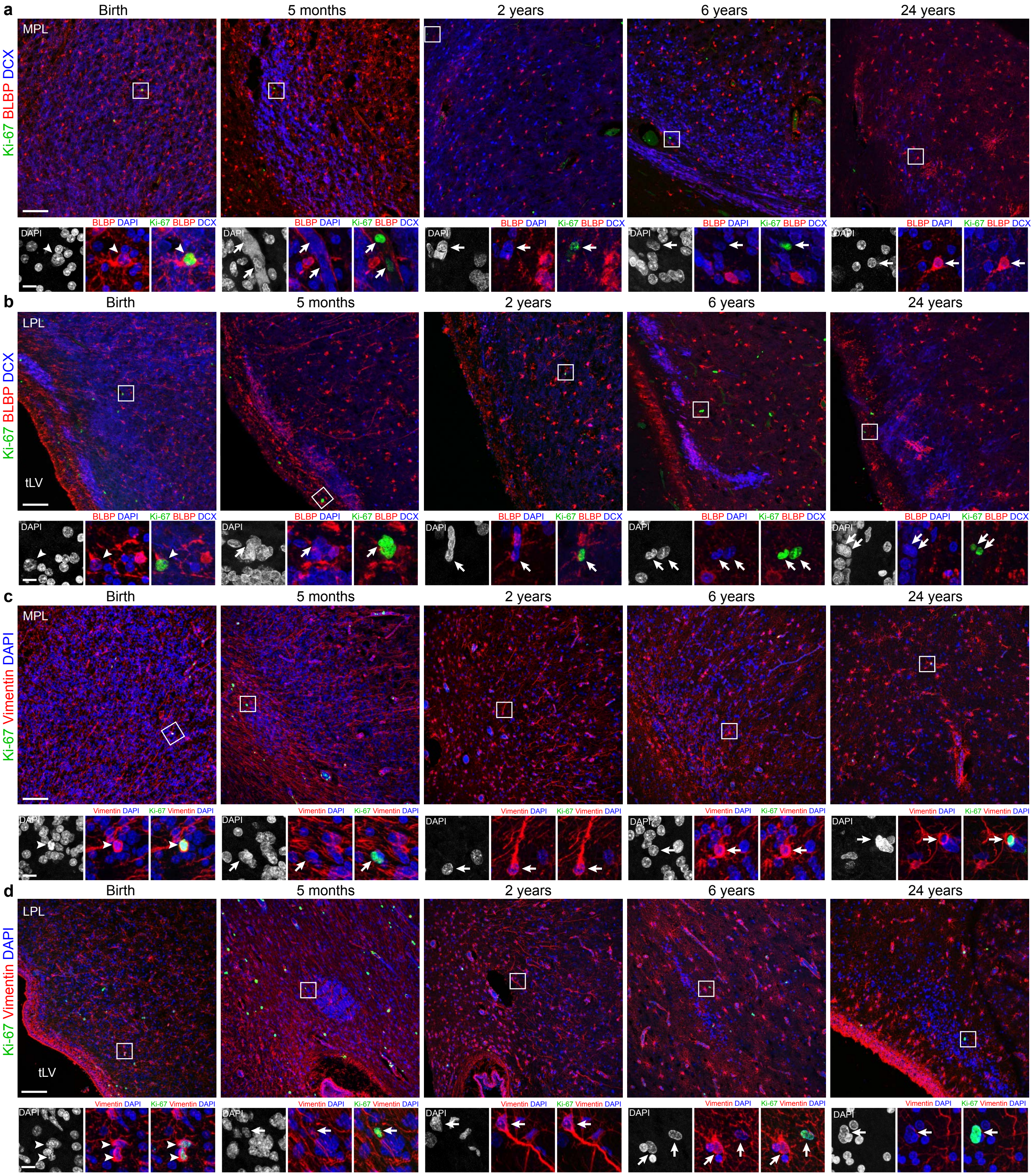


**Supplementary Figure 6: Ki-67⁺BLBP⁺ and Ki-67⁺vimentin⁺ cells in the PL decline between birth and 5 months. a,** Ki-67⁺BLBP⁺ cells in the MPL at birth (arrowheads), and single-positive Ki-67⁺ BLBP⁻ or Ki-67⁻ BLBP⁺ cells (arrows) in ages 5 months and older. **b,** Ki-67⁺BLBP⁺ cells in the LPL at birth (arrowheads), and single-positive Ki-67⁺BLBP⁻ or Ki-67⁻BLBP⁺ cells (arrows) in ages 5 months and older. **c,** Ki-67⁺vimentin⁺ cells in the MPL at birth (arrowheads), and single-positive Ki-67⁺vimentin⁻ or Ki-67⁻vimentin⁺ cells (arrows) in ages 5 months and older. **d,** Ki-67⁺vimentin⁺ cells in the LPL at birth (arrowheads), and single-positive Ki-67⁺vimentin⁻ or Ki-67⁻vimentin⁺ cells (arrows) in ages 5 months and older. Scale bars: 100 µm (**a–d** top), 10 µm (**a–d** bottom).

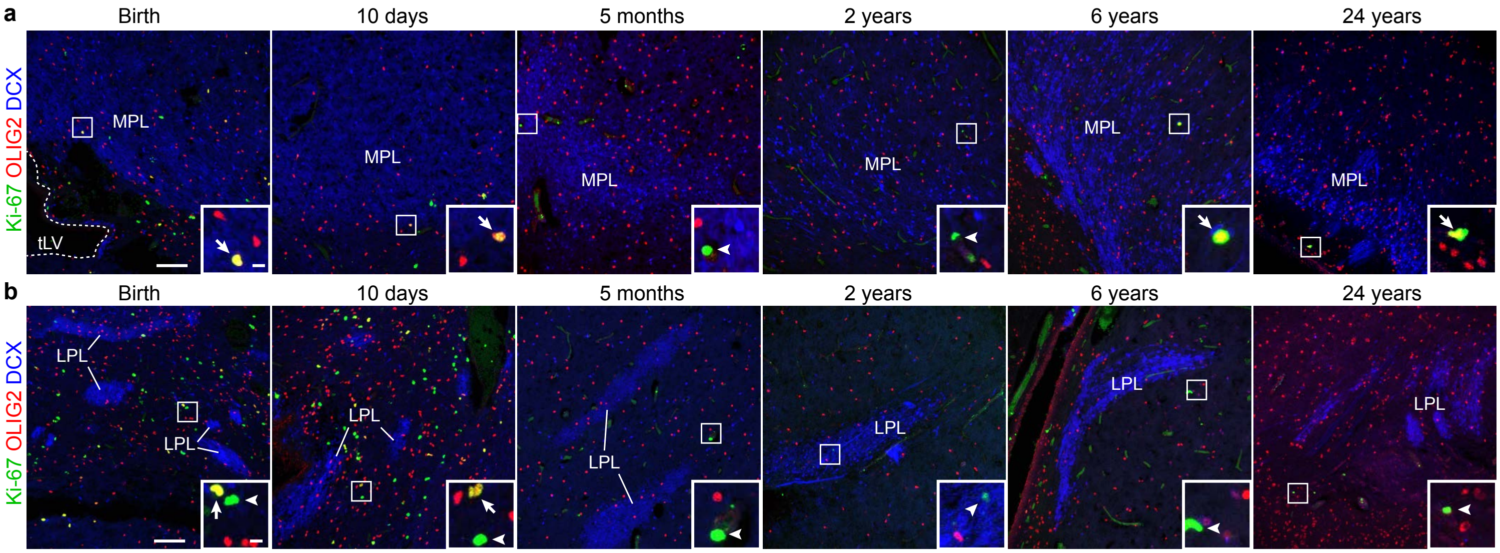


**Supplementary Figure 7: Ki-67⁺ OLIG2⁺ cells decline in the postnatal PL. a,b,** Ki-67$^+$OLIG2$^+$ cells (arrows) and Ki-67$^+$OLIG2$^-$ cells (arrowheads) in the MPL (**a**) and LPL (**b**) from birth to 24 years of age. Scale bars: 100 µm (**a**,**b**), 10 µm (insets).

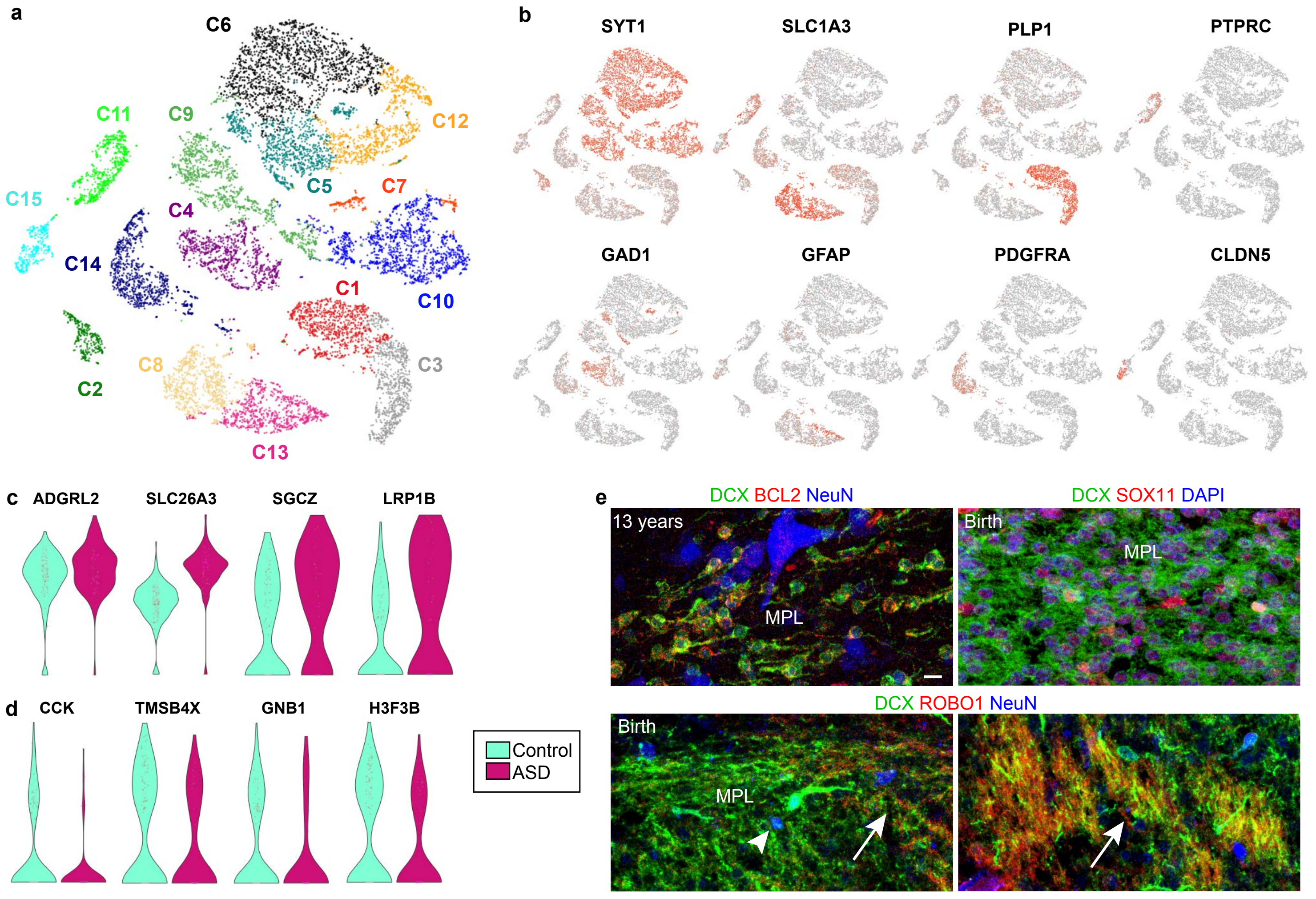


**Supplementary Figure 8: Clustering of snRNA-seq data from human amygdala and ASD-associated gene expression changes. a,** 15 cell clusters identified though unbiased clustering of single nuclei gene expression profiles. **b,** Markers of neuronal and glial cell types in the human amygdala snRNA-seq dataset. **c,d,** ASD-associated differentially expressed genes in immature PL neurons (cluster C2). **e,** Immunofluorescent validation of different markers identified in C2: DCX$^+$BCL2$^+$ cells in the MPL at 13 years of age, DCX$^+$SOX11$^+$ cells at birth, and DCX$^+$ processes that are ROBO1$^+$ at birth (arrows) near DCX$^+$ cell bodies that are ROBO1$^-$. Scale bar: 10 μm (**e**).

Supplementary Table 1- Case list

| Case no. | Age | Gender | PMI | Experimental Use | Neuropathology diagnosis | Clinical history |
|---|---|---|---|---|---|---|
| 1 | 20 GW | M | 42h | 1,2 | control | spontaneous abortion |
| 2 | 21 GW | F | 14h | 1,2 | control | osteogenesis imperfecta |
| 3 | 22 GW | M | 24h | 1–3,9 | control | urethral stenosis |
| 4 | 22 GW | M | 48h | 1–3,9 | control | spontaneous abortion |
| 5 | 26 GW | F | 24h | 1,2 | control | spontaneous abortion |
| 6 | 34 GW | F | 42h | 1,2 | control | lung malformation |
| 7 | 37 GW | M | 48h | 6,8 | control | VATER malformation |
| 8 | 38 GW | M | 50h | 1–7,9 | control | bronchopulmonary dysplasia |
| 9 | 39 GW | F | 48h | 3,4 | control | intrauterine demise |
| 10 | 39 GW | M | 24h | 3,4 | control | congenital diaphragmatic hernia |
| 11 | term | M | 14h | 1–7 | control | chondrodysplasia |
| 12 | term | M | 11h | 1–3,5,7 | control | cardiac dysfunction |
| 13 | term | M | 5h | 1–7 | control | respiratory failure |
| 14 | term | M | 24h | 1–7 | control | hydronephrosis |
| 15 | 1 day | F | 36h | 1–3,5,7 | control | renal hypoplasia; hypoplastic lung |
| 16 | 2 day | M | 36h | 1–7,11 | control | hypoplastic L heart |
| 17 | 10 days | F | 19h | 10 | control | respiratory failure |
| 18 | 1 month | M | 10h | 2 | control | congenital renal agenesis |
| 19 | 1.5 months | F | 21h | 1–7 | control | congenital diaphragmatic hernia |
| 20 | 3 months | M | 16h | 1–7 | control | diaphragmatic hernia |
| 21 | 3 months | M | 20h | 10 | control | diaphragmatic hernia |
| 22 | 5 months | M | 44h | 1–7,10 | control | congenital diaphragmatic hernia |
| 23 | 6 months | M | 14h | 7 | control | VATER malformation |
| 24 | 1 year | F | 48h | 6,7 | control | right pulmonary vein stenosis |
| 25 | 2 years | M | 19h | 1–7,10 | control | leukemia |
| 26 | 3 years | M | 25h | 7 | control | esophageal atresia, tracheoesophageal fistula |
| 27 | 6 years | F | 36h | 1–8,10 | control | congenital left diaphragmatic hernia |
| 28 | 6 years | M | 16h | 7,12 | control | drowning |
| 29 | 7 years | M | 3h | 7,12 | ASD | complications of cancer |
| 30 | 7 years | M | 21h | 11 | control | septic shock from acute appendicitis |
| 31 | 8 years | M | 12h | 7,12 | ASD | drowning |
| 32 | 8 years | M | 12h | 7,12 | control | drowning |
| 33 | 11 years | M | 27h | 7,12 | ASD | acute hemorrhagic tracheobronchitis |
| 34 | 12 years | M | 13h | 7,12 | control | drowning |
| 35 | 13 years | M | 12h | 3–9 | control | focal segmental glomerulosclerosis |
| 36 | 15 years | M | 9h | 7,12 | control | cardiac arrhythmia due to conduction system |
| 37 | 15 years | F | 13h | 7,12 | ASD | drowning associated with seizure disorder |
| 38 | 15 years | M | 22h | 7,8 | control | acute lymphoblastic leukemia |
| 39 | 19 years | M | 48h | 10 | control | systemic fungal infection |
| 40 | 24 years | F | 16h | 1–8,10 | control | acute kidney injury, multiorgan failure |
| 41 | 36 years | M | 56h | 7 | control | interstitial lung disease |
| 42 | 45 years | M | 2h | 11 | control | pleural cancer |
| 43 | 48 years | M | 5h | 11 | control | autoimmune nephritis w/ transplant |
| 44 | 49 years | F | 23h | 7,8 | control | undifferentiated round cell sarcoma |
| 45 | 55 years | M | 36h | 7 | control | heart failure |
| 46 | 57 years | F | 9h | 10 | control | papillary thyroid carcinoma |
| 47 | 67 years | F | 19h | 10 | control | chronic obstructive lung disease |
| 48 | 77 years | M | 50h | 7,8 | control | VHL with hemangiomas |
| 49 | 78 years | F | 23h | 7 | control | hypertension, history of aortic dissection |

**Key to experimental use:**

| | |
|---|---|
| 1 Ki-67;SP8;COUPTFII;PROX1 | 7 DCX;PSA-NCAM;NeuN |
| 2 DCX;COUPTFII;NeuN | 8 DCX;CB |
| 3 Ki-67;SOX2;DCX | 9 DCX;TBR1 |
| 4 Ki-67;BLBP;Vimentin | 10 DCX; interneurons |
| 5 Ki-67;DCX;PSA-NCAM | 11 DCX; immunogold |
| 6 Ki-67;Olig2;DCX | 12 snSeq |

Supplementary Table 2 - Antibodies

| Antigen | Species | Dilution | Manufacturer | Cat. No. | Antigen Retrieval | Lot No. | Manufacturer Specificity Description |
|---|---|---|---|---|---|---|---|
| ALDH1L1 | Mouse | 1:500 | NeuroMab | N103/39 | None | N103/31 | Human reactivity |
| BCL2 | Mouse | 1:100 | Santa Cruz Biotech | sc-7382 (C-2) | 10 min | K1218 | Raised against human Bcl-2 |
| BLBP | Rabbit | 1:200 | EMD Millipore | ABN14 | 10 min | 2299161 | Human reactivity predicted based on sequence |
| BLBP | Mouse | 1:200 | Abcam | ab131137 | 10 min | AT1D1 | Human reactivity |
| Calbindin | Rabbit | 1:1000 | Swant | CB-38a | 10 min | 9.03 | Human reactivity |
| Calretinin | Mouse | 1:1000 | Swant | 6B3 | 10 min | 7699/4 | Human reactivity |
| CoupTFII | Mouse | 1:250 | R&D Systems | PP-H7147-00 | 10 min | A-2 | Raised against human COUPTFII |
| Doublecortin | Rabbit | 1:200 | Cell Signaling | 4604S | None | 42798 | Human reactivity |
| Doublecortin | Guinea pig | 1:200 | EMD Millipore | AB2253 | None | 2787730 | Human reactivity predicted based on sequence |
| GFAP | Chicken | 1:750 | Abcam | ab4674 | None | GR267558-1 | Human reactivity |
| Iba1 | Rabbit | 1:100 | Wako | 019-1974 | None | LKJ2979 | Reactivity to human Iba1 |
| Ki-67 | Mouse | 1:200 | BD Pharmigen | 556003 | 10 min | 6110925 | QC resting: human |
| Ki-67 | Rabbit | 1:500 | Novocastra | NCL-Ki67p | 10 min | 6029714 | Specificity: human Ki-67 |
| Ki-67 | Rabbit | 1:1000 | Vector Labs | VP-K451 | None | 6013873 | Specificity: human Ki-67 |
| MAP2 | Chicken | 1:500 | Abcam | ab5392 | None | GR286806-6 | Human reactivity |
| Nestin | Mouse | 1:250 | Covance | MMS-570p | None | 14683401 | Human reactivity |
| NeuN | Chicken | 1:500 | EMD Millipore | ABN91 | None | 2620673 | Human reactivity |
| NeuN | Rabbit | 1:1000 | Novus Biologicals | R-3770-100 | None | 201605-SH | Raised against human FOX3 |
| Neurofilament | Mouse | 1:1000 | Abcam | ab24574 | None | GR191433-7 | Human reactivity |
| NKX2.1 | Rabbit | 1:500 | Santa Cruz Biotech | sc-13040 (H-190) | 10 min | B2216 | Raised against human NKX2.1 |
| nNOS | Rabbit | 1:500 | EMD Millipore | AB5380 | None | 2519293 | Raised against human nNOS |
| NPY | Rabbit | 1:500 | Abcam | ab30914 | None | GR212905-1 | Human reactivity |
| OLIG2 | Rabbit | 1:750 | EMD Millipore | AB9610 | None | 2519344 | Human reactivity |
| PROX1 | Rabbit | 1:500 | EMD Millipore | AB5475 | 8 min | LV1354325 | Human reactivity |
| PROX1 | Goat | 1:500 | R&D Systems | AF2727 | 10 min | VIY0216011 | Human reactivity |
| PSA-NCAM | Mouse | 1:1000 | EMD Millipore | MAB5324 | None | 2201402 | Human reactivity |
| ROBO1 | Mouse | 1:100 | Santa Cruz Biotech | sc-293444 | 10 min | D2817 | Raised against human ROBO1 |
| SCGN | Rabbit | 1:1000 | Sigma-Aldrich | HPA006641 | None | A106808 | Human reactivity |
| SOX2 | Goat | 1:200 | Santa Cruz Biotech | sc-17320 (Y-17) | 10 min | H2914 | Reactivity to human SOX2 |
| SOX2 | Rabbit | 1:200 | Cell Signaling | 2748S | 10 min | 2 | Reactivity to human SOX2 |
| SOX11 | Rabbit | 1:500 | EMD Millipore | AB5776 | 10 min | 3054693 | Human reactivity |
| SP8 | Goat | 1:200 | Santa Cruz Biotech | sc-104661 (C-18) | 8 min | G0516 | Human reactivity |
| SST | Goat | 1:250 | Santa Cruz Biotech | sc-7819 (D-20) | None | G0716 | Human reactivity |
| TBR1 | Chicken | 1:200 | EMD Millipore | AB2261 | None | 2893188 | Human reactivity |
| TUJ1 | Mouse | 1:200 | Covance | MMS-435P | 10 min | TU1 | Human reactivity |
| VGLUT2 | Guinea pig | 1:200 | EMD Millipore | AB5907 | None | 2894024 | Rat (protein seq. 98% identical to human) |
| Vimentin | Mouse | 1:1000 | Sigma-Aldrich | V5255 | None | 045K4826 | Tested in human appendix/ tonsil |

Supplementary Table 3 - Genes differentially expressed in immature PL neurons in ASD

| gene.IDs | gene.names | gene.biotypes | FCs | FDRs | FC_raw |
|---|---|---|---|---|---|
| ENSG00000091138 | SLC26A3 | protein_coding | 5.850149658 | 0.0001871921422 | 1.847488373 |
| ENSG00000168702 | LRP1B | protein_coding | 1.922913347 | 0.001339982591 | 1.232924153 |
| ENSG00000138670 | RASGEF1B | protein_coding | 1.801840449 | 0.001572375975 | 1.299544569 |
| ENSG00000184305 | CCSER1 | protein_coding | 4.217426847 | 0.006826692191 | 0.4825503699 |
| ENSG00000205542 | TMSB4X | protein_coding | -1.233572225 | 0.01281857797 | -0.4848494181 |
| ENSG00000150756 | FAM173B | protein_coding | 0.2820599983 | 0.01721292848 | 0.1579495104 |
| ENSG00000187094 | CCK | protein_coding | -2.478100813 | 0.01873110543 | -0.521808086 |
| ENSG00000117114 | ADGRL2 | protein_coding | 0.3843599182 | 0.02164326496 | 0.3808905808 |
| ENSG00000275443 | RP11-759A24.3 | lincRNA | -0.9530566461 | 0.02723474619 | -0.165065828 |
| ENSG00000078369 | GNB1 | protein_coding | -0.5159267986 | 0.02827392498 | -0.3942154221 |
| ENSG00000111011 | RSRC2 | protein_coding | -2.144482041 | 0.02848930642 | -0.3959280793 |
| ENSG00000105649 | RAB3A | protein_coding | -2.54553859 | 0.02903743238 | -0.265926041 |
| ENSG00000128989 | ARPP19 | protein_coding | -1.422262201 | 0.03169490743 | -0.5170711773 |
| ENSG00000100554 | ATP6V1D | protein_coding | -2.254287096 | 0.03182949225 | -0.2666499763 |
| ENSG00000179889 | PDXDC1 | protein_coding | -1.108149114 | 0.03182949225 | -0.4178134235 |
| ENSG00000185053 | SGCZ | protein_coding | 1.308263765 | 0.03562456606 | 1.148732649 |
| ENSG00000164919 | COX6C | protein_coding | -2.477741609 | 0.03570725607 | -0.1755408476 |
| ENSG00000136267 | DGKB | protein_coding | 0.9101420031 | 0.03619039185 | 0.8408377592 |
| ENSG00000154174 | TOMM70A | protein_coding | 0.804467377 | 0.03619039185 | 0.3046166414 |
| ENSG00000179104 | TMTC2 | protein_coding | 0.7391567743 | 0.03619039185 | 0.700715308 |
| ENSG00000236824 | BCYRN1 | lincRNA | 0.2701744597 | 0.03619039185 | 0.6806952491 |
| ENSG00000132475 | H3F3B | protein_coding | -1.036045754 | 0.03712499988 | -0.5241241606 |
| ENSG00000175395 | ZNF25 | protein_coding | -2.129270255 | 0.03782169224 | -0.1595052421 |
| ENSG00000232573 | RPL3P4 | processed_pseudogene | -0.8582949501 | 0.03782169224 | -0.3671730924 |
| ENSG00000070785 | EIF2B3 | protein_coding | -3.267738217 | 0.03821687899 | -0.309978585 |
| ENSG00000126457 | PRMT1 | protein_coding | -2.335274072 | 0.03821687899 | -0.1931841097 |
| ENSG00000145416 | MARCH1 | protein_coding | 1.075637619 | 0.03892016793 | 0.7278414853 |
| ENSG00000147684 | NDUFB9 | protein_coding | -1.242078339 | 0.03960912211 | -0.1711299453 |
| ENSG00000151376 | ME3 | protein_coding | -2.634126749 | 0.04435063816 | -0.5051282709 |
| ENSG00000152784 | PRDM8 | protein_coding | -2.441363985 | 0.04585269919 | -0.188538258 |
| ENSG00000170396 | ZNF804A | protein_coding | 0.7594812117 | 0.04585269919 | 0.6881329164 |
| ENSG00000259589 | RP11-317G6.1 | antisense | -2.502210026 | 0.04648736138 | -0.1641248921 |
| ENSG00000114784 | EIF1B | protein_coding | -1.474803654 | 0.04881442074 | -0.5132531336 |
