# Supplementary material for: Immature excitatory neurons develop during adolescence in the human amygdala
Source: Nat Commun. 2019 Jun 21;10:2748. doi: 10.1038/s41467-019-10765-1 (PMC6588589; doi:10.1038/s41467-019-10765-1)
Supplement: Supplementary file 3 — Description of Additional Supplementary Files [file 41467_2019_10765_MOESM3_ESM.pdf]

Description of Additional Supplementary Files

File Name: Supplementary Data 1
Description: Mixed linear models

File Name: Supplementary Data 2
Description: Cell metadata for the amygdala snRNA-seq dataset

File Name: Supplementary Data 3
Description: List of markers of immature PL neurons identified by unbiased analysis of snRNA-seq data
