# Supplementary material for: Immature excitatory neurons develop during adolescence in the human amygdala
Source: Nat Commun. 2019 Jun 21;10:2748. doi: 10.1038/s41467-019-10765-1 (PMC6588589; doi:10.1038/s41467-019-10765-1)
Supplement: Supplementary file 7 — Reporting Summary [file 41467_2019_10765_MOESM7_ESM.pdf]

natureresearch

Corresponding author(s): Arturo Alvarez-Buylla

Last updated by author(s): Apr 4, 2019

Reporting Summary

Nature Research wishes to improve the reproducibility of the work that we publish. This form provides structure for consistency and transparency in reporting. For further information on Nature Research policies, see Authors & Referees and the Editorial Policy Checklist.

Statistics

For all statistical analyses, confirm that the following items are present in the figure legend, table legend, main text, or Methods section.

n/a	Confirmed	
☐	☒	The exact sample size (*n*) for each experimental group/condition, given as a discrete number and unit of measurement
☐	☒	A statement on whether measurements were taken from distinct samples or whether the same sample was measured repeatedly
☐	☒	The statistical test(s) used AND whether they are one- or two-sided<br>*Only common tests should be described solely by name; describe more complex techniques in the Methods section.*
☒	☐	A description of all covariates tested
☐	☒	A description of any assumptions or corrections, such as tests of normality and adjustment for multiple comparisons
☐	☒	A full description of the statistical parameters including central tendency (e.g. means) or other basic estimates (e.g. regression coefficient) AND variation (e.g. standard deviation) or associated estimates of uncertainty (e.g. confidence intervals)
☐	☒	For null hypothesis testing, the test statistic (e.g. *F*, *t*, *r*) with confidence intervals, effect sizes, degrees of freedom and *P* value noted<br>*Give P values as exact values whenever suitable.*
☒	☐	For Bayesian analysis, information on the choice of priors and Markov chain Monte Carlo settings
☒	☐	For hierarchical and complex designs, identification of the appropriate level for tests and full reporting of outcomes
☒	☐	Estimates of effect sizes (e.g. Cohen's *d*, Pearson's *r*), indicating how they were calculated

Our web collection on statistics for biologists contains articles on many of the points above.

Software and code

Policy information about availability of computer code

Data collection	MBF Biosciences Neurolucida, Leica LASAF, ImageJ
Data analysis	MBF Biosciences Neurolucida, ImageJ, GraphPad Prism, R

For manuscripts utilizing custom algorithms or software that are central to the research but not yet described in published literature, software must be made available to editors/reviewers. We strongly encourage code deposition in a community repository (e.g. GitHub). See the Nature Research guidelines for submitting code & software for further information.

Data

Policy information about availability of data

All manuscripts must include a data availability statement. This statement should provide the following information, where applicable:

- Accession codes, unique identifiers, or web links for publicly available datasets
- A list of figures that have associated raw data
- A description of any restrictions on data availability

Raw RNA-seq data (fastq files) are available at Sequence Read Archive (SRA), accession number PRJNA530977. The authors declare that the data supporting the findings of this study are available within the paper and its supplementary information files.

Field-specific reporting

Please select the one below that is the best fit for your research. If you are not sure, read the appropriate sections before making your selection.

☒ Life sciences ☐ Behavioural & social sciences ☐ Ecological, evolutionary & environmental sciences

Life sciences study design

All studies must disclose on these points even when the disclosure is negative.

Sample size	We initially assessed the variance of each cell population quantified within four age groups: gestation (20 GW to 39 GW; n=10), infancy (term to 1 year; n = 14), childhood and adolescence (2 to 19 years; n = 7), and adulthood (24 to 78 years; n = 9). These data were used to conduct power analyses which guided section sampling frequency and total numbers of cells evaluated for marker expression.
Data exclusions	No data were excluded.
Replication	Experiments were repeated at minimum in technical triplicate; all attempts at replication were successful.
Randomization	The tissue samples at each age were not allocated into experimental groups, so randomization did not apply.
Blinding	Investigators were blinded to the age of the tissue sample during image collection and during quantification.

Reporting for specific materials, systems and methods

We require information from authors about some types of materials, experimental systems and methods used in many studies. Here, indicate whether each material, system or method listed is relevant to your study. If you are not sure if a list item applies to your research, read the appropriate section before selecting a response.

Materials & experimental systems

n/a	Involved in the study
☐	☒ Antibodies
☒	☐ Eukaryotic cell lines
☒	☐ Palaeontology
☒	☐ Animals and other organisms
☒	☐ Human research participants
☒	☐ Clinical data

Methods

n/a	Involved in the study
☒	☐ ChIP-seq
☒	☐ Flow cytometry
☒	☐ MRI-based neuroimaging

Antibodies

Antibodies used

Antigen Manufacturer Cat. No. Lot No.
ALDH1L1 NeuroMab N103/39 N103/31
BCL2 Santa Cruz Biotech sc-7382 (C-2) K1218
BLBP EMD Millipore ABN14 2299161
BLBP Abcam ab131137 AT1D1
Calbindin Swant CB-38a 9.03
Calretinin Swant 6B3 7699/4
CoupTFII R&D Systems PP-H7147-00 A-2
Doublecortin Cell Signaling 4604S 42798
Doublecortin EMD Millipore AB2253 2787730
GFAP Abcam ab4674 GR267558-1
Iba1 Wako 019-1974 LKJ2979
Ki-67 BD Pharmigen 556003 6110925
Ki-67 Novocastra NCL-Ki67p 6029714
Ki-67 Vector Labs VP-K451 6013873
MAP2 Abcam ab5392 GR286806-6
Nestin Covance MMS-570p 14683401
NeuN EMD Millipore ABN91 2620673
NeuN Novus Biologicals R-3770-100 201605-SH
Neurofilament Abcam ab24574 GR191433-7
NKX2.1 Santa Cruz Biotech sc-13040 (H-190) B2216
nNOS EMD Millipore AB5380 2519293
NPY Abcam ab30914 GR212905-1
OLIG2 EMD Millipore AB9610 2519344
PROX1 EMD Millipore AB5475 LV1354325
PROX1 R&D Systems AF2727 VIY0216011
PSA-NCAM EMD Millipore MAB5324 2201402
ROBO1 Santa Cruz Biotech sc-293444 D2817
SCGN Sigma-Aldrich HPA006641 A106808
SOX2 Santa Cruz Biotech sc-17320 (Y-17) H2914
SOX2 Cell Signaling 2748S 2

SOX11 EMD Millipore AB5776 3054693
SP8 Santa Cruz Biotech sc-104661 (C-18) G0516
SST Santa Cruz Biotech sc-7819 (D-20) G0716
TBR1 EMD Millipore AB2261 2893188
TUJ1 Covance MMS-435P TU1
VGLUT2 EMD Millipore AB5907 2894024
Vimentin Sigma-Aldrich V5255 045K4826

Validation

Antigen Manufacturer Specificity Description
ALDH1L1 Human reactivity
BCL2 Raised against human Bcl-2
BLBP Human reactivity predicted based on sequence
BLBP Human reactivity
Calbindin Human reactivity
Calretinin Human reactivity
CoupTFII Raised against human COUPTFII
Doublecortin Human reactivity
Doublecortin Human reactivity predicted based on sequence
GFAP Human reactivity
Iba1 Reactivity to human Iba1
Ki-67 QC resting: human
Ki-67 Specificity: human Ki-67
Ki-67 Specificity: human Ki-67
MAP2 Human reactivity
Nestin Human reactivity
NeuN Human reactivity
NeuN Raised against human FOX3
Neurofilament Human reactivity
NKX2.1 Raised against human NKX2.1
nNOS Raised against human nNOS
NPY Human reactivity
OLIG2 Human reactivity
PROX1 Human reactivity
PROX1 Human reactivity
PSA-NCAM Human reactivity
ROBO1 Raised against human ROBO1
SCGN Human reactivity
SOX2 Reactivity to human SOX2
SOX2 Reactivity to human SOX2
SOX11 Human reactivity
SP8 Human reactivity
SST Human reactivity
TBR1 Human reactivity
TUJ1 Human reactivity
VGLUT2 Rat (protein seq. 98% identical to human)
Vimentin Tested in human appendix/ tonsil

Conditions of use for each antibody was validated by the manufacturer. We evaluated each antibody by comparison to no primary antibody (negative) controls, comparison to (often enriched) expression in human gestational tissue, and comparison to rodent staining patterns.
